# Unilateral Compressive Optic Neuropathy As the Presenting Manifestation of Clival Chordoma: A Case Report

**DOI:** 10.7759/cureus.24440

**Published:** 2022-04-24

**Authors:** Francesco Pellegrini, Daniele Brocca, Alessandra Cuna, Altin Stafa, Andrew G Lee

**Affiliations:** 1 Ophthalmology, Santo Spirito Hospital, Pescara, ITA; 2 Ophthalmology, De Gironcoli Hospital, Conegliano, ITA; 3 Radiology, Ca Foncello Hospital, Treviso, ITA; 4 Ophthalmology, Houston Methodist Hospital, Houston, USA

**Keywords:** brain tumor, optic neuropathy, clivus, clival chordoma, compressive optic neuropathy

## Abstract

The optic nerve(s) may be compressed by a number of intracranial and intraorbital masses. Compression may be isolated to the optic nerve or may involve other intracranial or intraorbital structures with variable presentation. A 26-year-old man presented with complaints of progressive painless visual loss in the right eye for eight months. Examination revealed isolated unilateral optic atrophy consistent with a diagnosis of compressive optic neuropathy. Magnetic resonance imaging of the brain showed compression of the anterior visual pathways due to a lesion radiologically compatible with clival chordoma. He underwent subtotal neurosurgical resection and pathology was consistent with chordoma. Although rare, isolated unilateral visual loss may be the only presenting manifestation of clival chordoma.

## Introduction

Clival chordoma (CC) is a rare neoplasm that arises from remnants of the embryonic notochord. Common sites of chordoma development are the sacrococcygeal area and the skull base. Intracranial chordomas usually arise from the region of the clivus. Diplopia is a common complaint but isolated optic nerve compression is rare; among intracranial neoplasms, chordomas were found in less than 0.7% of cases. We describe a case of visual loss due to unilateral compressive optic neuropathy as the presenting manifestation of this uncommon intracranial tumor. 

## Case presentation

**Figure 1:**
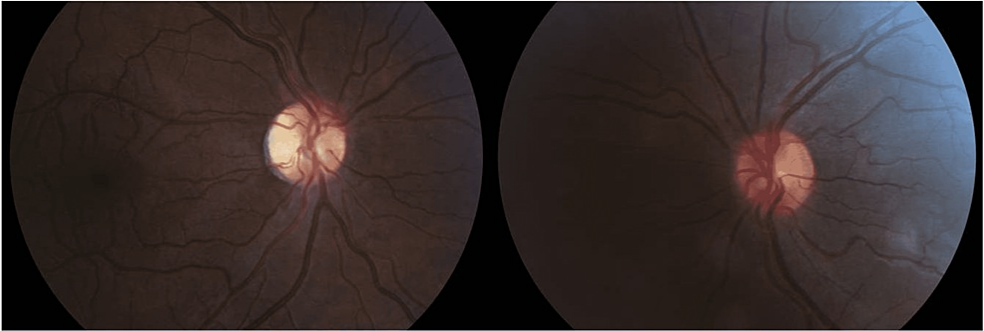
Retinography is normal in the left eye (right image) while shows optic disc pallor in the right eye (left image)

A 26-year-old man presented complaining of progressive visual loss in the right eye (OD) for the past eight months. He denied headache, diplopia, or any other neurologic symptoms and felt otherwise well. His past medical history was non-contributory and he was taking no medications. Best-corrected visual acuity (BCVA) was 20/200 OD and 20/20 in the left eye (OS). Extraocular motility was full. External exam was normal and there was no proptosis or ptosis. Pupils were isocoric with a right relative afferent pupillary defect (RAPD). Intraocular pressure measurements and anterior segment examination were within normal limits in both eyes (OU). Fundus examination (Figure 1) revealed optic nerve pallor OD and a normal optic disc OS.

**Figure 2:**
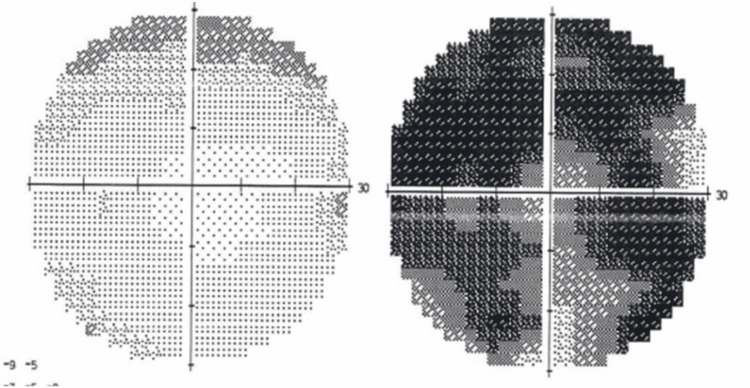
Standard computerized visual field 30-2 shows diffuse defect OD (right) with a superior initial fascicular defect OS (left). OD: oculus dexter (right eye); OS: oculus sinister (left eye)

**Figure 3:**
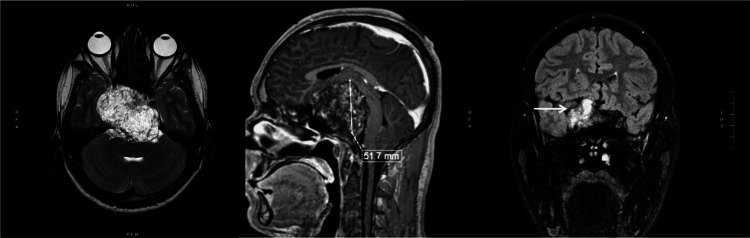
Brain MRI: (1) extreme left is T2-weighted axial fat saturation image showing a large intracranial tumor in the region of the clivus; (2) Middle image is post contrast T1-weighted sagittal view showing the large (51.7 mm height) tumor displacing superiorly the midbrain and the chiasm region; (3) Extreme right is T2-weighted coronal FLAIR image showing the tumor (arrow) extending to the right side. FLAIR: Fluid-attenuated inversion recovery

**Figure 4:**
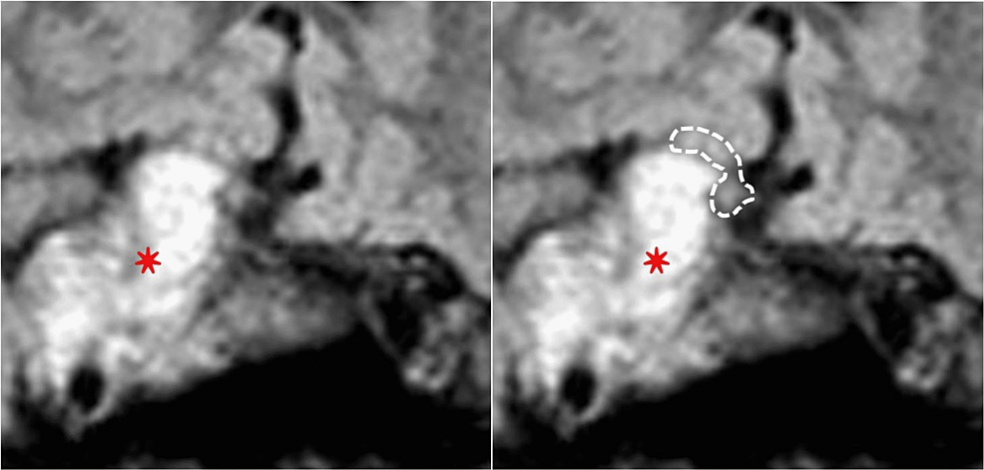
Brain MRI: High magnification view in the chiasmatic region showing the tumor (red star) displacing the chiasm (dotted line on the right) upward and asymmetrically compressing it on the right.

**Figure 5:**
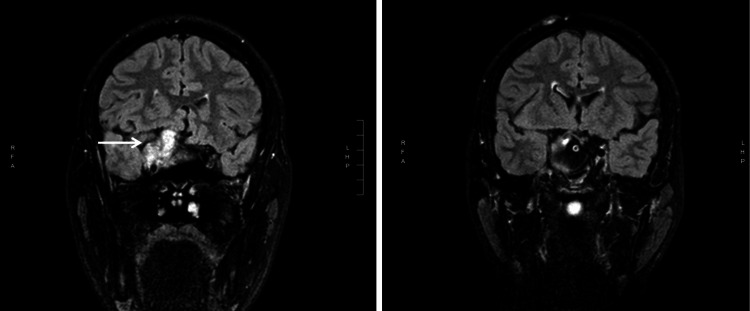
Brain MRI: T2-weighted coronal FLAIR pre-surgery (left image) and post-surgery (right image) FLAIR: Fluid-attenuated inversion recovery

The standard computerized visual field (Figure 2) showed a diffuse defect OD and a superior mild fascicular defect OS. Magnetic resonance imaging (MRI) of the brain showed a large intracranial mass with radiologic features of CC (Figure 3), with the upward displacement of the chiasm and asymmetric involvement of optic nerves (figure 4). The patient underwent subtotal neurosurgical resection and pathology was consistent with a chordoma (Figure 5) but there was no improvement in visual acuity or visual field.

## Discussion

CC is a rare neoplasm with an incidence rate of less than 0.1 per 100.000 per year [1]. CCs arise from remnants of the embryonic notochord [2] and show both epithelial and mesenchymal differentiation. Common sites of chordoma development are the sacrococcygeal area in 50-60% of cases and the skull base (spheno-occipital area) in 25-35% of the cases. Uncommonly, it may develop from cervical, thoracic, or lumbar vertebrae [3].

Histologically chordomas show a typical pattern of lobules separated by fibrous bands. Each lobule displays vacuolated, atypical neoplastic cells within a myxoid stroma [4]. Age at presentation is around the fifth or sixth decades for the sacrococcygeal type and even earlier for skull-base chordomas [3].

In several large reviews of patients with intracranial neoplasms, chordomas were found in 0.1-0.7% of patients [5]. Intracranial chordomas usually arise from the region of the clivus and account, as previously reported, for about one-third of all cases [3]. Although the tumor rarely metastasizes, advanced secondary lesions may affect the lungs, bone, liver soft tissues, lymph nodes, and skin [6]. When arising from the clivus, common symptoms are headache, facial numbness, nasal discharge, dysphagia, and cranial nerve palsy. Diplopia is a common complaint since the most common cranial nerve to be compressed is the sixth cranial nerve with unilateral or bilateral abducens deficit. Third and fourth nerve palsies are also possible as well as complete unilateral ophthalmoplegia from multiple cranial nerve involvement. 

Volpe and coworkers reviewed the neuro-ophthalmologic findings in chordomas and chondrosarcomas of the skull base [5]. In the group of 48 patients with chordoma, decreased visual acuity was present in only four patients (8%) but in only two cases (4%) was visual loss an isolated finding. The pattern of the visual field loss differed in each patient, with some displaying a central scotoma, altitudinal defects, junctional scotoma, or bi-temporal hemianopsia. In a series of 12 patients, Harbour and coworkers found that visual loss was present in only three patients (25%), but none of them complained of visual loss as the presenting symptom [7]. In another case series of 63 patients affected by intracranial chordoma, Bagan et al. found 39 patients (62%) with isolated ophthalmic manifestations [8]. Visual loss was present in only 10 patients (16%) and among those patients who initially had only one symptom, only three of them (4.7%) complained of isolated visual loss.

## Conclusions

Although isolated and progressive unilateral visual loss from compression of the anterior visual pathway is an uncommon finding in CC, clinicians should be aware of this presentation. Neuroimaging typically demonstrates the compressive lesion and the origin at the clivus consistent with CC. Gross total resection is the best treatment.
